# Anosmia in COVID-19: Investigating the Role of Paranasal Sinus Mucosal Thickening

**DOI:** 10.7759/cureus.56019

**Published:** 2024-03-12

**Authors:** Vijayalakshmi Sampath, Pradeebaa Thiyagarajan, Thivakaran Tamilarasan, Suhasini Balasubramaniam, Shanmuga Ashok Sivaramakrishnan, Vijay Sathish Kumar Irulappan, Ishwar Gopinath, Suresh Kumar Rajamal, Rupert Nithin Fernando, Swaminathan Ramasubramanian

**Affiliations:** 1 Radiodiagnosis, Government Medical College, Omandurar Government Estate, Chennai, IND; 2 Radiodiagnosis, Government RSRM (Raja Sir Ramaswamy Mudaliar) Hospital, Chennai, IND; 3 Radiodiagnosis, Government Stanley Medical College and Hospital, Chennai, IND; 4 Otolaryngology - Head and Neck Surgery, Government Medical College, Omandurar Government Estate, Chennai, IND; 5 Pathology, Government Medical College, Omandurar Government Estate, Chennai, IND; 6 Radiodiagnosis, Davao Medical School Foundation, Davao, PHL

**Keywords:** ct pns, mucosal thickening, paranasal sinus, anosmia, covid-19

## Abstract

Background

Anosmia has been identified as a distinctive symptom of COVID-19, leading to hypotheses about its pathophysiological underpinnings, including the potential role of paranasal sinus mucosal thickening.

Objective

To investigate the association between paranasal sinus mucosal thickening and anosmia in COVID-19 patients, providing insights into the complex clinical manifestations of the disease.

Methods

This retrospective cohort study analyzed CT paranasal sinus from 270 confirmed COVID-19 patients, divided into those with anosmia (n = 23, 8.52%) and those without anosmia (n = 247, 91.48%). Statistical analysis, including independent t-tests, was employed to compare mucosal thickening between the groups.

Results

The study found an average mucosal thickening of 0.03 in patients with anosmia and 0.02 in those without, with no statistically significant difference between the groups (p = 0.480, which is greater than 0.05). The findings suggest that mucosal thickening in the paranasal sinuses does not serve as a definitive correlate of anosmia among COVID-19 patients.

Conclusion

The absence of a significant correlation between paranasal sinus mucosal thickening and anosmia in COVID-19 patients indicates that the pathophysiology of anosmia may involve factors beyond anatomical changes, including direct viral effects and systemic inflammatory responses.

## Introduction

The recent global health crisis, instigated by the emergence of the severe acute respiratory syndrome coronavirus 2 (SARS-CoV-2) virus in Wuhan, China, toward the end of 2019, has unveiled a plethora of clinical manifestations extending beyond the conventional respiratory symptoms associated with coronaviruses. Among these, anosmia, or the loss of smell, has emerged as a distinctive and prevalent symptom of coronavirus disease 2019 (COVID-19), marking a significant deviation from the symptomatology of viral respiratory infections. This unique clinical presentation, particularly when occurring in the absence of other respiratory symptoms like nasal congestion, has not been traditionally linked to such infections, thereby presenting a diagnostic anomaly in the context of the pandemic [1]. As COVID-19 spread across continents, affecting millions, the medical community rapidly recognized the diverse symptomatology of the disease, with anosmia becoming a hallmark symptom for many patients. This phenomenon prompted a reevaluation of the impact of the virus on human physiology, leading to hypotheses that ventured beyond direct viral invasion of respiratory pathways. Notably, the theory that mucosal thickening in the paranasal sinuses (PNS) might obstruct or alter olfactory neuron signaling emerged as a significant area of interest. Such alterations in the mucosa could potentially disrupt the olfactory pathways, providing a plausible explanation for the anosmia observed in some COVID-19 cases [2-5].

Further research into the pathophysiological underpinnings of COVID-19-related anosmia has revealed a complex landscape of potential mechanisms. Initial studies focused on the direct effects of the virus on the olfactory system, including the olfactory epithelium and bulb, as well as its impact on central nervous system pathways. These investigations have suggested a range of viral effects that could lead to the sudden onset of anosmia, highlighting the multifaceted nature of SARS-CoV-2's impact on the body [6,7]. However, the involvement of PNS mucosal thickening in the pathophysiology of anosmia has been less clear. Imaging studies aimed at assessing mucosal changes in the PNS of COVID-19 patients have produced mixed findings. Several studies have raised questions about the role of sinus pathology in COVID-19-related anosmia [8]. Some reports emphasize viral effects on the olfactory system, downplaying mucosal thickening in the nasal or sinus regions [9]. In light of the lack of significant mucosal thickening in many cases, alternative explanations have been explored. These include subtler PNS changes impacting olfactory function, such as inflammation affecting receptor function or signal transduction. Moreover, systemic inflammation and immune responses to COVID-19 may also indirectly influence anosmia, adding complexity to its understanding [10,11].

By supplementing the existing body of knowledge, this study aims to clarify the potential pathophysiological origins of anosmia concerning mucosal changes in the PNS, contributing to a more comprehensive understanding of the diverse clinical presentations of COVID-19.

## Materials and methods

Study design and setting

This retrospective cohort study was conducted in the Department of Radiodiagnosis at Government Medical College, Omandurar Government Estate, Chennai, affiliated to the Tamil Nadu Dr. M.G.R. Medical University designated as a COVID-19 Care Center during the initial phase of the pandemic. The study involved the comprehensive review of medical records and imaging findings, specifically computed tomography scans of paranasal sinuses (CT PNS), of patients diagnosed with COVID-19 and admitted to this tertiary care hospital. Institutional Ethics Committee approval was obtained, as denoted by the reference IEC No. 23/IEC/GOMC/2021, ensuring adherence to the ethical guidelines of the institutional research committee and the 1964 Helsinki Declaration and its later amendments or comparable ethical standards.

Study period

Data were collected over three months, from April to June 2021, coinciding with a significant increase in COVID-19 cases, providing a timely and relevant dataset for analysis.

Participants

Adult patients, aged 18 years and older, who were hospitalized during the study period, comprised the research cohort. Informed consent was obtained through telephonic interviews, leveraging contact information from medical records. The cohort was stratified into two groups: Group A, which included patients experiencing anosmia, and Group B, which consisted of those who did not report this symptom.

Inclusion criteria

The study established a set of inclusion criteria designed to carefully select patients for analysis, focusing on adult individuals presenting with clinical manifestations of COVID-19 and confirmed diagnosis through reverse transcription-polymerase chain reaction (RT-PCR) testing during their hospitalization. Participants were required to be 18 years or older, capturing a demographic capable of providing informed consent. Eligibility extended to those exhibiting a range of clinical symptoms indicative of COVID-19, including fever, cough, and difficulty breathing, among others, to ensure a comprehensive representation of the clinical spectrum of the disease [12]. A confirmed positive COVID-19 RT-PCR test result during the hospital stay was imperative for inclusion. Additionally, only patients with complete medical records and available CT PNS imaging data were considered, enabling a thorough investigation of the impact of COVID-19 on the paranasal sinuses.

Exclusion criteria

Patients under 18 years of age, those lacking clinical symptoms of COVID-19 [12], or those without a confirmed positive RT-PCR test for COVID-19 were excluded from the study to maintain the focus on a clearly defined patient population with confirmed diagnoses.

Sample size

A total of 270 patients meeting the inclusion criteria were selected through convenience sampling, providing a substantial dataset for analysis.

CT imaging protocol

The CT imaging protocol was meticulously designed to ensure consistency and reliability across all patient scans. The procedure utilized a 16-section multidetector CT scanner, specifically the Aquilion Lightning model TSX 035a by Toshiba (Tokyo, Japan), chosen for its precision in capturing detailed images. Patients underwent scanning in the supine position, with instructions to hold their breath during the procedure to minimize motion artifacts, thereby enhancing image quality. Image analysis was performed using Vitrea software version 6.5.99 (Vital Images, Inc., Ōtawara, Japan), recognized for its advanced diagnostic capabilities. The scans were conducted with specific technical parameters, including a voltage of 120 kV, a current of 180 mA, a rotation time of 0.5 seconds, a pitch of 0.531, and a section collimation of 0.62 mm, aimed at optimizing the balance between image clarity and patient safety. An axial fusion-compliant data set with a 22 cm field of view was generated for each patient, focusing on the quantitative evaluation of mucosal thickening in the paranasal sinuses. Two independent radiologists with a combined 17 years of experience, blind to the patients' clinical backgrounds, reviewed the imaging. Any discrepancies were resolved through discussion or consultation with a third expert radiologist, ensuring a high level of accuracy and consensus in the imaging analysis.

Data management

Mucosal thickening in CT imaging was subjected to quantitative assessment on a scale ranging from zero to one. The ethmoidal air cells were arbitrarily partitioned into four segments, with each segment allocated a value of 0.25 in the presence of discernible mucosal presence interfacing between the air cavity and the surrounding bony structure. Data from CT images and patient records were meticulously entered into a secure Microsoft Excel (Microsoft Corporation, Redmond, WA) spreadsheet, with stringent confidentiality measures in place to protect patient privacy.

Statistical analysis

The statistical analysis of the study was conducted using Python version 3.9 (Python Software Foundation, Wilmington, DE), leveraging its powerful and versatile computing capabilities. The analysis utilized several key libraries, including *pandas* for data manipulation and analysis, *NumPy* for numerical operations, *SciPy* for more advanced statistical functions, and *Matplotlib* for generating graphs and visualizations. This combination of tools allowed for the efficient handling and analysis of the data, enabling the computation of descriptive statistics for each group and the application of independent t-tests to compare the extent of mucosal thickening between the groups with and without anosmia. A p-value of less than 0.05 was set as the threshold for statistical significance, adhering to standard practices in medical research.

Ethical considerations

The study was conducted adhering to the ethical guidelines of our institution, ensuring the confidentiality and anonymity of patient data. To ensure anonymity, patient identification information was removed from the data using picture archiving and communication system (PACS) software. Unique random numbers were then assigned to each image and clinical data record to allow linking while maintaining anonymity. Data were securely stored in the computer system of the Department of Radiodiagnosis in a password-protected folder. All procedures performed in studies involving human participants were in accordance with the ethical standards of the institutional and/or national research committee.

## Results

**Figure 1:**
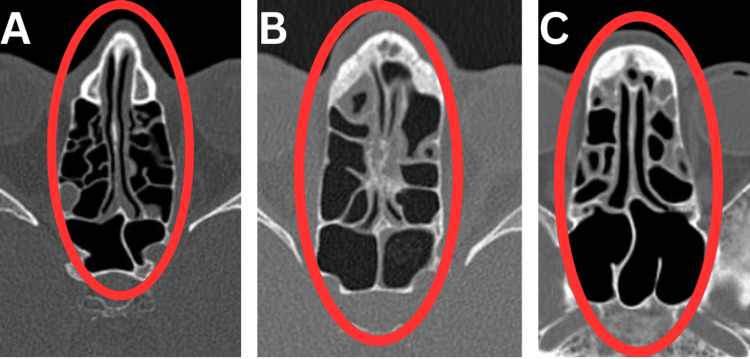
Axial non-contrast computed tomography (CT) images of the paranasal sinuses (PNS) highlighting the extent of sinus involvement. (A) Opacification of less than 25% of the ethmoid air cells is seen. (B) Opacification of 25-50% of the ethmoid air cells is seen. (C) Opacification of 50-75% of the ethmoid air cells is seen. Red ovals highlight the ethmoidal air cells, with the degree of sinus involvement estimated visually.

A comprehensive analysis of mucosal thickening in the paranasal sinuses, as observed in CT PNS images, was performed on a cohort of 270 confirmed COVID-19 patients. Figure 1 shows CT images with varying mucosal thickening.

Upon scrutinizing the data from the total cohort (Table 1), it was noted that the average mucosal thickening stood at 0.02. The observed range for thickening spread between 0 and 0.75, with a standard deviation of 0.089875. Interestingly, both the median and mode values for the overall group were pegged at 0, suggesting that a substantial portion of patients did not experience any detectable mucosal thickening.

**Table 1:** Mucosal thickening in patients with and without anosmia. Average, range, standard deviation, median, and mode are for the level of mucosal thickening in computed tomography scans of paranasal sinuses.

Group	Number of patients (percentage)	Average	Range	Standard deviation	Median	Mode
Overall	270 (100%)	0.02	0-0.75	0.089875	0	0
Patients with anosmia	23 (8.52%)	0.03	0-0.25	0.087813	0	0
Patients without anosmia	247 (91.48%)	0.02	0-0.75	0.095021	0	0

A measure of inter-rater reliability, Cohen's kappa coefficient, was employed for this purpose. The kappa value was found to be 0.86, suggesting an "almost perfect" agreement between the two radiologists. From the subgroup of patients reporting anosmia (n = 23, 8.58%), the average mucosal thickening was found to be slightly elevated at 0.03. However, the range of thickening was narrower, spanning 0 to 0.25. The standard deviation in this group (0.087813) was marginally lower than the overall group, hinting at lesser variability among anosmic patients in terms of mucosal thickening.

In the larger cohort of patients who did not report anosmia (n = 247, 91.48%), the findings mirrored that of the overall group. The average mucosal thickening was consistent at 0.02. The variation, denoted by the range of 0-0.75 and a standard deviation of 0.095021, was broader in this subgroup. Similar to the anosmia group and the overall cohort, the median and mode were both at 0.

**Figure 2:**
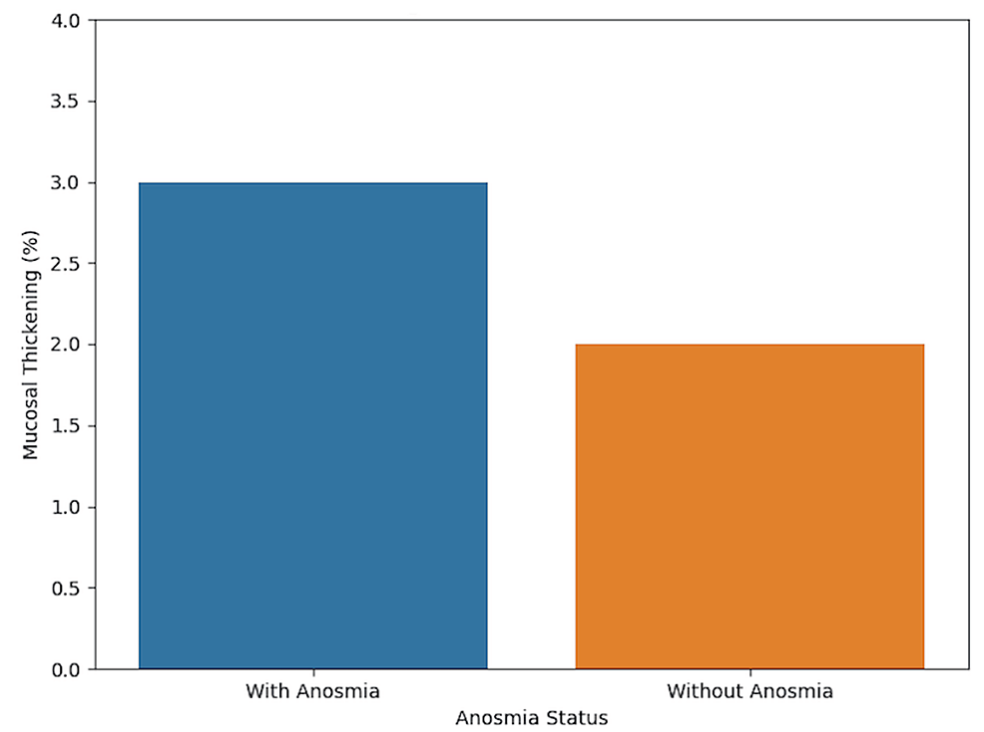
Bar graph showing 3% mucosal thickening for patients with anosmia and 2% for those without anosmia. Average mucosal thickening in patients with anosmia was at 0.03, which is represented as 3%, and in patients without anosmia was at 0.02, which is represented as 2%.

Figure 2 illustrates a bar graph of 0.03 (3%) mucosal thickening for patients with anosmia versus 0.02 (2%) for those without anosmia.

Violin plot analysis

**Figure 3:**
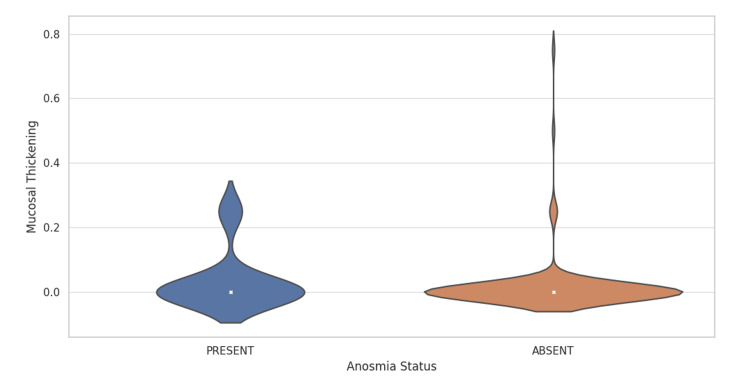
Violin plot showing the distribution of mucosal thickening for both "PRESENT" and "ABSENT" anosmia statuses.

The violin plot (Figure 3) provides a visual summary of the mucosal thickening distributions across patients with and without anosmia. For patients presenting anosmia, the mucosal thickening values are predominantly clustered near zero with fewer cases extending up to 0.75, suggesting a narrower spread. In contrast, patients without anosmia exhibit a similar distribution but with a slightly wider spread and an increased density of cases with no mucosal thickening. The thicker sections of the violin plot indicate a higher density of data points, which in this case, are concentrated near the zero mark for both groups.

Density plot analysis

**Figure 4:**
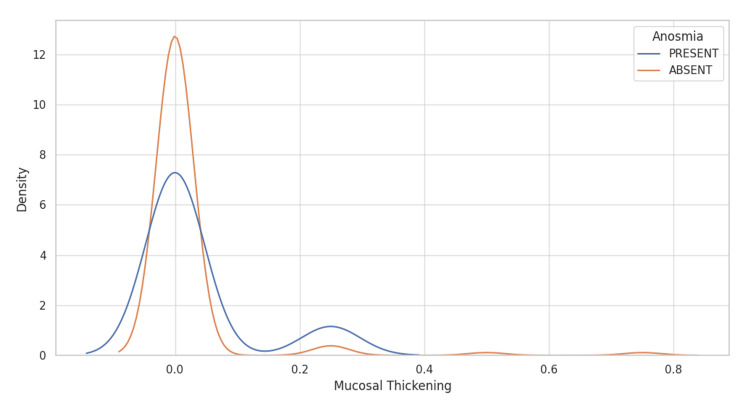
Density plot showing the density of mucosal thickening, differentiated by anosmia status. The x-axis represents mucosal thickening and the y-axis represents the probability density of values, reflecting the distribution shape and spread of the mucosal thickening measurements.

The density plot (Figure 4) illustrates the probability density of the mucosal thickening values for the two anosmia patient groups. Both groups demonstrate a peak at 0.0 mucosal thickening, with a rapid fall-off as the values increase. This indicates that a majority of patients, regardless of their anosmia status, have low to no mucosal thickening. The tails of the distribution for both groups suggest a few cases with higher mucosal thickening values. Notably, the density plot suggests that the presence or absence of anosmia does not significantly alter the distribution of mucosal thickening values.

Together, these visualizations provide insights into the distribution characteristics of mucosal thickening among patients stratified by their anosmia status. The consistency in the concentration of lower mucosal thickening values across both groups may suggest a limited impact of anosmia status on mucosal thickening.

An independent t-test was used to compare mucosal thickening between the two groups. The result (t = 0.70696) suggested that the means of the two groups were not statistically different. This was further validated by a p-value of 0.480, surpassing the standard significance threshold of 0.05.

While the average mucosal thickening in patients with anosmia was slightly higher at 0.03 (3%) compared to those without anosmia at 0.02 (2%), the statistical evidence did not support a significant difference. This was consistent across median and mode values for both groups. Taken together, the data suggest that while mucosal thickening in the paranasal sinuses is evident among COVID-19 patients, it does not serve as a definitive correlate of anosmia. The robust agreement between radiologists further adds to the credibility of these findings.

## Discussion

In evaluating the intricate association between anosmia and mucosal thickening within the paranasal sinuses in individuals afflicted by COVID-19, our study embarked on a meticulous assessment of CT PNS images. This methodological approach was validated by the high degree of consensus among interpreting radiologists, achieving an "almost perfect" kappa value, underscoring the reliability of our findings. Despite the thoroughness of our analysis, the data yielded a rather subtle connection between the extent of mucosal thickening and the manifestation of anosmia among COVID-19 patients. Although a higher incidence of mucosal thickening was observed in patients experiencing anosmia compared to those without this symptom, the disparity did not reach statistical significance. Our findings contribute to an ongoing discourse within the scientific community regarding the relationship between COVID-19 and paranasal sinus involvement. For instance, the work of Sumi et al. examined mucosal thickening across different sinus regions, uncovering significant variability and demonstrating that SARS-CoV-2-positive patients did not exhibit markedly different mucosal thickening scores from those uninfected by the virus [13]. This observation suggests that the presence of SARS-CoV-2 alone does not necessarily predispose individuals to increased mucosal thickening in the sinuses. Similarly, Keshavarz et al. highlighted that the majority of COVID-19-related anosmia cases did not present with notable paranasal sinus abnormalities on CT scans [14], further questioning the direct correlation between sinonasal mucosal changes and anosmia.

Contrary to the limited evidence linking mucosal thickening directly with anosmia, other research avenues have unearthed compelling findings regarding the olfactory system's involvement. Studies have documented olfactory cleft opacification, alterations in olfactory bulb volume, appearance, and signal intensity among COVID-19 patients [5]. These radiologic signatures suggest a significant impact of the virus on the olfactory apparatus, potentially elucidating the pathogenic mechanisms underlying anosmia. Naeini et al. proposed that the invasion of the olfactory epithelium by the virus, facilitated by the expression of the angiotensin-converting enzyme 2 (ACE2) receptor and TMPRSS2 enzyme in non-neural olfactory cells, might be a pivotal factor in the development of anosmia, offering a perspective that shifts the focus from sinonasal mucosal involvement to direct viral effects on olfactory tissues [15].

Our investigation aligns with existing literature in finding that severe mucosal thickening is not a hallmark of COVID-19, indicating a wide variation in mucosal thickening among affected individuals [13]. The specific analysis of anosmia patients revealed a marginally higher, albeit statistically insignificant, level of mucosal thickening compared to non-anosmic individuals, suggesting a complex interplay between mucosal thickening and anosmia that may not solely be attributed to structural changes detectable by imaging. This complexity is further highlighted by contrasting studies, which challenge the direct correlation between detectable mucosal changes and anosmia, suggesting that the loss of smell in COVID-19 could stem from factors beyond anatomical alterations observable in imaging studies [15]. The literature also raises concerns about the exacerbation of mucosal thickening through invasive fungal infections, such as mucormycosis, in susceptible COVID-19 patients [16], indicating a critical area for future inquiry, particularly given the severe outcomes associated with such infections. The physiological roles of the paranasal sinuses, including the humidification and heating of inhaled air, may intersect with observed clinical presentations in COVID-19 patients, offering insights into the pathophysiological impact of the virus on these structures. The association of COVID-19 with acute rhinosinusitis, attributed to fungal species like *Aspergillus*, further complicates the relationship between COVID-19 and sinus health, suggesting that mucosal thickening could also reflect a broader spectrum of sinusitis conditions [17].

Acknowledging the limitations inherent in our study is crucial for contextualizing its findings within the broader research landscape. The modest sample size, especially concerning patients experiencing anosmia, may limit the statistical robustness of our conclusions. The retrospective nature of the study introduces potential biases, including selection and information biases. Variabilities in CT PNS scanning protocols, equipment, and interpretation could have contributed to inconsistencies in data collection. The reliance on self-reported anosmia raises the possibility of recall bias, and the study's confinement to a single tertiary care center may restrict the generalizability of the findings. Additionally, factors such as pre-existing sinonasal conditions, prior anosmia history, or other respiratory diseases were not fully accounted for, and the timing of CT scans relative to the onset of illness was not standardized, potentially influencing the observed mucosal changes. These considerations underscore the necessity for future research to adopt a larger, prospective, multicentric approach to elucidate the nuances of this relationship more definitively.

Our study underscores the nuanced and multifaceted nature of the relationship between paranasal sinus mucosal thickening and anosmia in COVID-19 patients. The absence of a statistically significant difference in mucosal thickening between anosmic and non-anosmic patients in our cohort calls for a more detailed exploration into the mechanisms underlying anosmia, which may extend beyond the simple anatomical changes to include direct viral effects and other pathophysiological processes. Drawing from the collective insights of our study and the wider body of literature, it is evident that COVID-19 exerts a complex impact on the paranasal sinuses, with broader implications for understanding and managing related complications, including anosmia. Future research endeavors should embrace a multidisciplinary approach, delving into the interplay between viral pathogenesis, host immune responses, and secondary complications, to unravel the comprehensive effects of COVID-19 on the paranasal sinuses and guide the development of nuanced therapeutic interventions.

## Conclusions

Our study reveals that mucosal thickening in the paranasal sinuses among COVID-19 patients does not significantly correlate with the presence of anosmia, suggesting the mechanisms behind anosmia extend beyond structural changes in the sinuses. This finding aligns with the broader understanding that COVID-19-related anosmia involves complex interactions involving direct viral effects, systemic inflammation, and multifactorial pathways, rather than merely anatomical modifications. It underscores the need for further investigation into the diverse clinical manifestations of COVID-19, highlighting the intricate interplay between the virus, host immune response, and various pathophysiological processes.
